# The Tnfaip8-PE complex is a novel upstream effector in the anti-autophagic action of insulin

**DOI:** 10.1038/s41598-017-06576-3

**Published:** 2017-07-24

**Authors:** Ji-Soo Kim, Jimin Park, Mi-Sun Kim, Ji-Young Ha, Ye-Won Jang, Dong Hae Shin, Jin H. Son

**Affiliations:** College of Pharmacy and Graduate School of Pharmaceutical Sciences, Global Top5 Research Program, Ewha W. University, Seoul, 03760 Republic of Korea

## Abstract

Defective hepatic autophagy is observed in obesity and diabetes, whereas autophagy is inhibited by insulin in hepatocytes. Insulin-induced anti-autophagy is mediated by non-canonical Gαi3 signaling *via* an unknown mechanism. Previously, we identified the anti-autophagic activity of Tnfaip8 *via* activation of mammalian target of rapamycin (mTOR) in the nervous system. Here, we demonstrate that insulin temporally induces Tnfaip8, which mediates the anti-autophagic action of insulin through formation of a novel ternary complex including Tnfaip8, phosphatidylethanolamine (PE) and Gαi3. Specifically, an X-ray crystallographic study of Tnfaip8 from *Mus musculus* (mTnfaip8) at 2.03 Å together with LC-MS analyses reveals PE in the hydrophobic cavity. However, an mTnfaip8 mutant lacking PE does not interact with Gαi3, indicating that the PE component is critical for the anti-autophagic action of mTnfaip8 *via* interaction with Gαi3. Therefore, the mTnfaip8-PE complex may act as an essential upstream effector *via* ternary complex formation most likely with active Gαi3 during insulin-induced anti-autophagy.

## Introduction

Autophagy is a homeostatic intracellular degradation process involving formation of double-membrane vacuoles (i.e., autophagosomes) and degradation in lysosomes^1^. Autophagy is stimulated during starvation, whereas it is inhibited by food intake. Defective hepatic autophagy underlies the hepatic insulin resistance observed in obesity and diabetes, which is normally regulated by insulin and/or amino acids^2^. Physiological inhibition of hepatic autophagy by insulin is mediated by the PI3K-Akt signaling pathway, leading to mammalian target of rapamycin (mTOR) activation^3^. Moreover, the Gαi3 subunit of the heterotrimeric G protein Gi3 is known to regulate enigmatic upstream crosstalk between the insulin receptor and PI3K-Akt-mTOR signaling during the anti-autophagic action of insulin in the liver^3, 4^. However, the detailed molecular mechanisms underlying non-canonical Gαi3-mediated signaling by insulin have not been fully elucidated.

Although members of the tumor necrosis factor (TNF)-alpha-induced protein 8 (Tnfaip8/TIPE/Oxi-α) family were originally described as regulators of tumorigenesis, inflammation and cell death^5, 6^, we previously discovered novel regulatory functions for these proteins in autophagy^7, 8^. For instance, Tnfaip8/TIPE/Oxi-α and Tnfaip8 l1/TIPE1/Oxi-β act as a pair of autophagy inhibitors and activators in Parkinson’s disease models^7, 8^. In particular, Tnfaip8 inhibits autophagy in an mTOR-dependent manner under oxidative stress conditions^7^. In accordance with our findings, the Drosophila Tnfaip8 homolog CG4091/sigmar was also found to be involved in autophagy activity during salivary gland development^9^.

The crystal structures of Tnfaip8l2/TIPE2/Oxi-γ (hTIPE2)^10^ and Tnfaip8l3/TIPE3/Oxi-δ (hTIPE3)^11^, two members of the Tnfaip8 family, from *Homo sapiens* have been determined. The structures both showed a central hydrophobic cavity occupied by two long electrostatic densities predicted to be phospholipid in nature. In particular, TIPE3 was reported to promote tumorigenesis by transferring phospholipids as second messengers^11^. However, the structural and molecular bases of Tnfaip8 in the process of autophagic inhibition remain to be elucidated.

This is the first report to elucidate the novel structure and function of mTnfaip8-phosphatidylethanolamine (PE) as an upstream effector in the anti-autophagic action of insulin, which occurs *via* ternary complex formation with Gαi3. In this study, we show that insulin treatment induces a temporal increase in mTnfaip8. X-ray crystallography and LC-MS (liquid chromatography-mass spectrometry) analyses reveal that mTnfaip8 is a novel PE-containing protein. Our findings demonstrate that the mTnfaip8-PE-Gαi3 ternary complex is essential for insulin-mediated anti-autophagy.

## Results

### Temporal induction of mTnfaip8 by insulin

**Figure 1:**
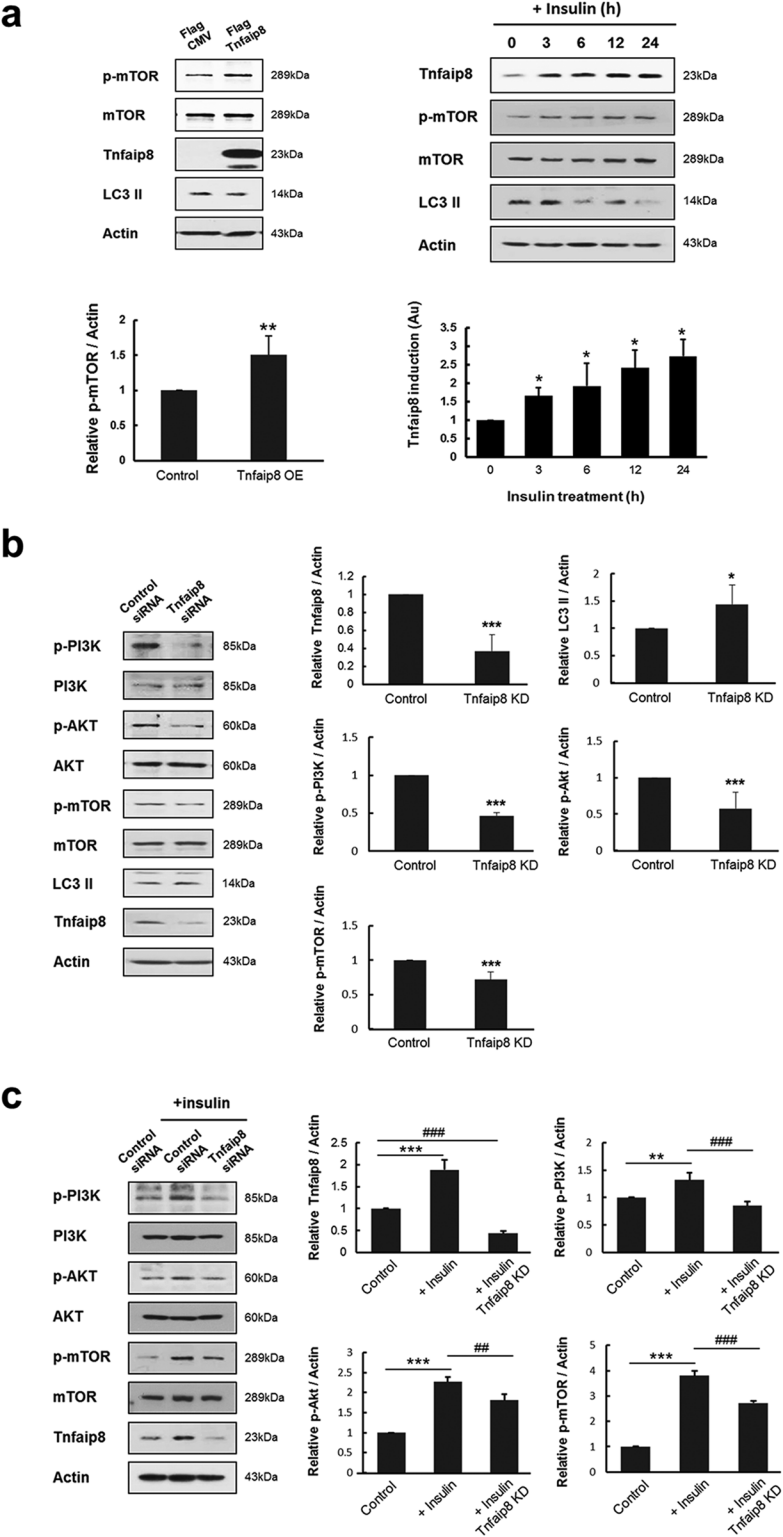
Temporal induction of mTnfaip8 by insulin. (**a**) mTnfaip8 was overexpressed in mouse hepatoma cells (Hepa 1−6) and phospho-mTOR and LC3 II levels were analyzed by immunoblotting after 48 h. The anti-autophagic activity of mTnfaip8 was confirmed (left panel). Hepa 1-6 cells were treated with insulin (1 µg/ml), and the levels of mTnfaip8 were analyzed by immunoblotting at 0 - 24 h. Insulin induced significant increases in mTnfaip8 levels over 24 h, which was accompanied by a significant decrease in the autophagy marker LC3 II (i.e., inhibition of autophagy) (right panel). (**b**,**c**) mTnfaip8 gene KD was performed in either absence or presence of insulin (1 µg/ml). The levels of phospho-PI3K, phospho-Akt and phospho-mTOR were analyzed by immunoblotting after 48 h. The levels of phospho-PI3K, phospho-Akt and phospho-mTOR were decreased significantly and autophagy was induced. Values are means (SD). *p < 0.05, ^##^p/**p < 0.01, ^###^p/***p < 0.001. The full-length blots are presented in Supplementary Figure 2 (Fig. S2).

Because mTnfaip8 and insulin are known to inhibit autophagy in neuronal and hepatic cells, respectively^3, 7^, we first investigated whether mTnfaip8 is regulated by insulin and acts as a potential effector molecule in the hepatic anti-autophagic action of insulin. The hepatic anti-autophagic activity of mTnfaip8 was first confirmed in a hepatic cell line as shown in Fig. 1a. When the temporal induction of mTnfaip8 by insulin was analyzed, insulin was found to induce an increase in mTnfaip8, with its increased levels of expression over 24 h (Fig. 1a). Moreover, this mTnfaip8 induction was accompanied by significant inhibition of autophagy, as demonstrated by a simultaneous decrease in the autophagy marker LC3 II (Fig. 1a). We next investigated whether PI3K-Akt signaling in anti-autophagic action of insulin is affected by mTnfaip8 gene knock down (KD) in either absence or presence of insulin. As expected, the levels of phospho-PI3K, phospho-Akt and phospho-mTOR were decreased significantly and the induction of autophagy was observed in both the absence and presence of insulin (Fig. 1b,c). These data suggest that mTnfaip8 may act as an upstream mediator of insulin-induced hepatic anti-autophagy via PI3K-Akt-mTOR signaling.

### The crystal structure of mTnfaip8 with a central hydrophobic cavity

**Figure 2:**
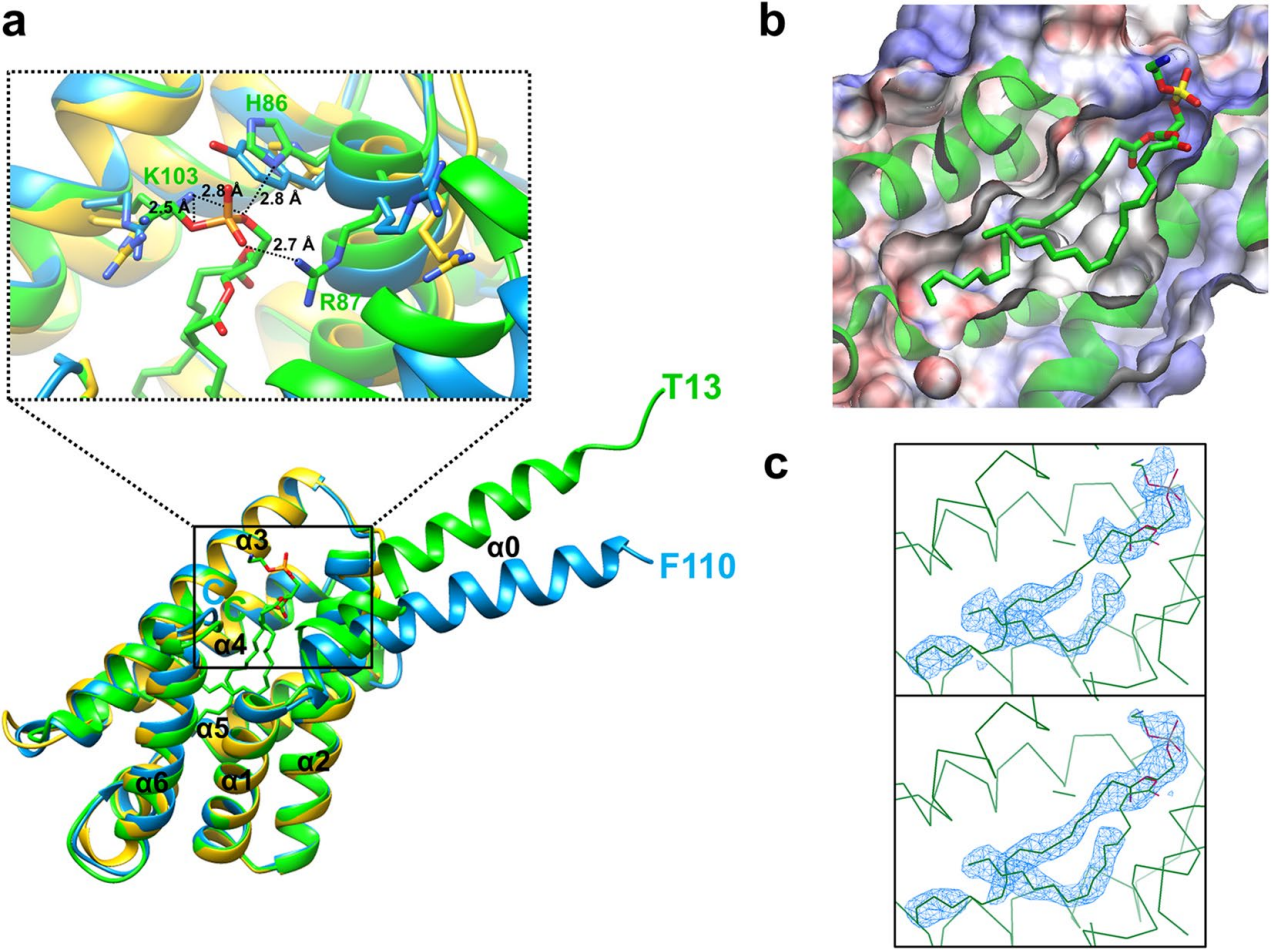
Structural analyses of mTnfaip8. (**a**) The superimposed crystal structure of mTnfaip8 (green) with that of hTIPE2 (gold; PDB ID: 3F4M) and hTIPE3 (sky blue; 4Q9V) are shown. The entrance of the cavity is highlighted in the solid box. The starting N-terminal residues are labeled according to their ribbon colors. The zoomed view represents the binding mode of PE in the highlighted region. (**b**) The hydrophobic cavity of mTnfaip8 with PE. (**c**) The composite omit map (upper) and the 2Fo-Fc electron density map (lower), contoured at the 0.9 σ level, for the region of PE are shown in blue mesh.

**Figure 3:**
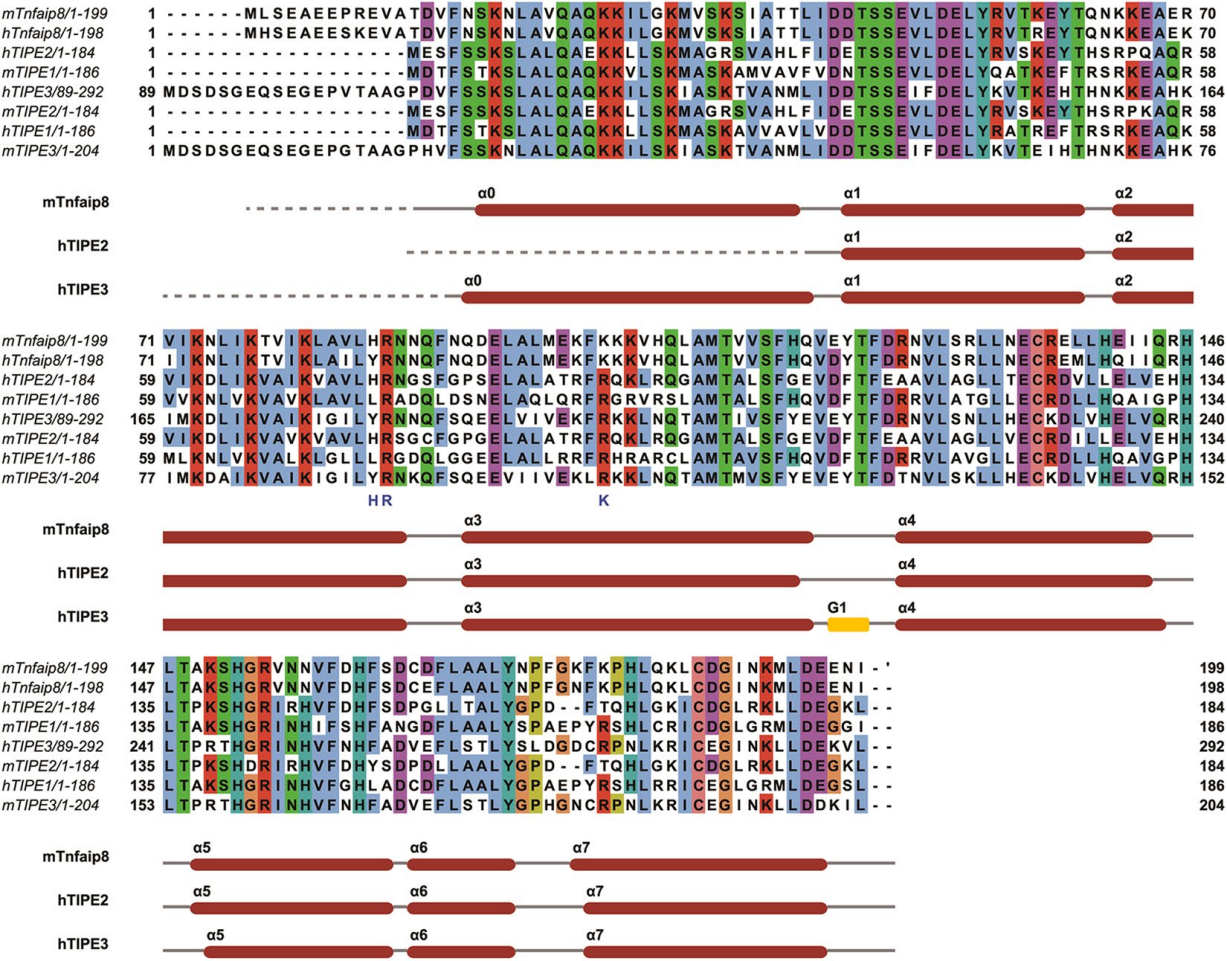
Sequence alignment of Tnfaip8 family members. Secondary structure elements of mTnfaip8, hTIPE2 and hTIPE3 are displayed and colored in red for α-helix, green for β-strand and yellow for 3_10_-helix. The blue characters represent key residues of mTnfaip8 for PE binding. Accession codes are BC009090.1 for Tnfaip8 from *Mus musculus* (mTnfaip8), AAH07014 for Tnfaip8 from *Homo sapiens* (hTnfaip8), BC032199.1 for TIPE1 from *Mus musculus* (mTIPE1), NP_001161414 for TIPE1 from *Homo sapiens* (hTIPE1), EDL38778 for mTIPE2 (*Mus musculus*), NP_078851 for TIPE2 from *Homo sapiens* (hTIPE2), NP_001028707 for TIPE3 from *Mus musculus* (mTIPE3), NP_997264 for TIPE3 from *Homo sapiens* (hTIPE3).

**Figure 4:**
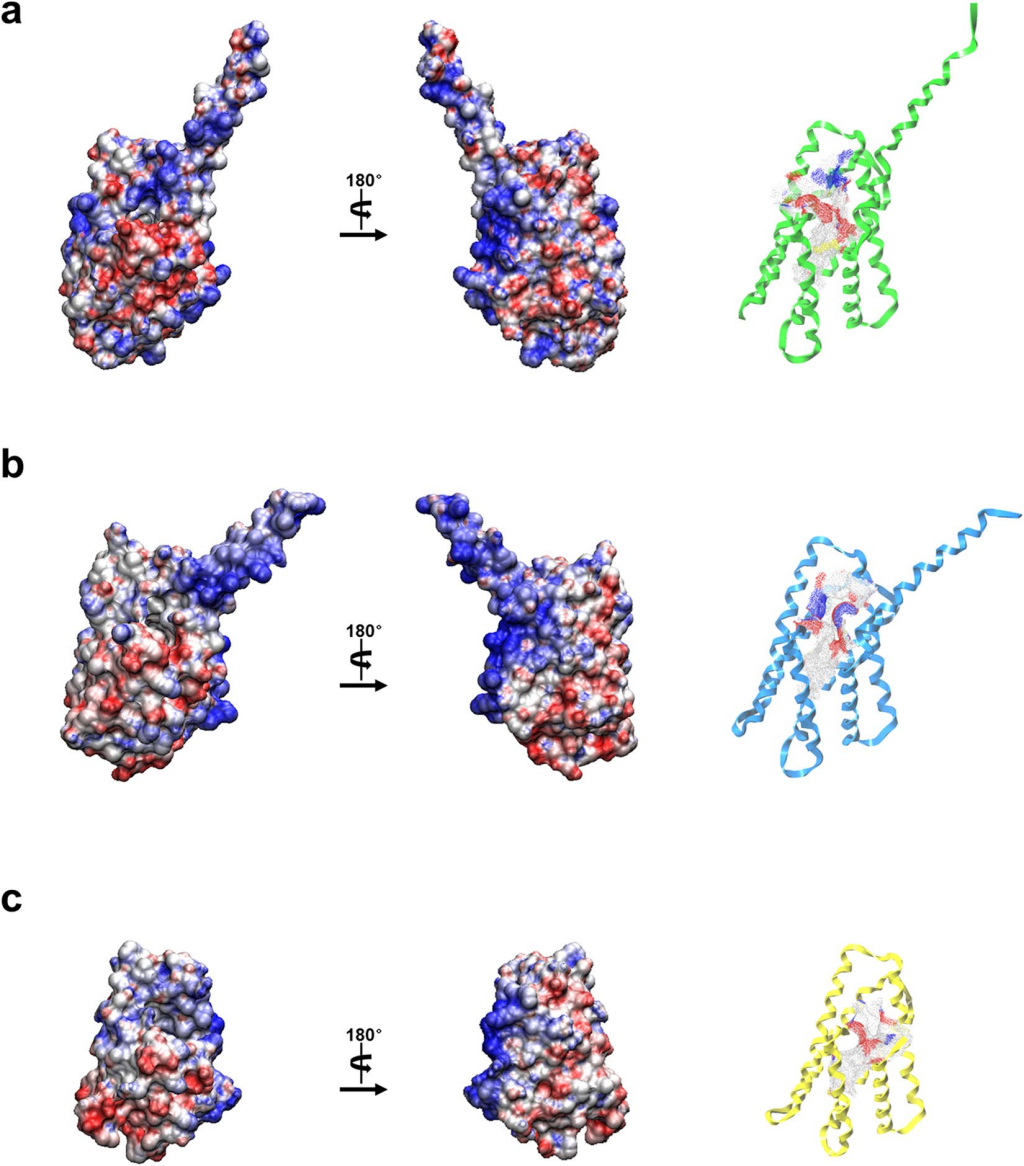
Structural comparison of (**a**) mTnfaip8, (**b**) hTIPE2 and (**c**) hTIPE3. The surface of the protein is drawn with an electrostatic surface model. Molecular surfaces were created by the VMD molecular-graphics software package^33^ following electrostatic calculations using APBS^34^ (red, negative; blue, positive; white, uncharged). The cavity is found and is depicted inside the ribbon diagram using the MOLCAD program of SYBYL-X2.0. The surface color of the cavity is represented according to the charge of residues comprising the cavity.

The crystal structure of Tnfaip8 from *Mus musculus* (mTnfaip8) was determined at 2.03 Å resolution (Table 1). Except for 12 flexible N-terminal residues (Met1-Ala12), all other amino acids are defined in the electron density map. The overall shape of mTnfaip8 resembles a water dipper. Its cylindrical domain has a dimension of 48 Å × 31 Å × 30 Å connected to an N-terminal grip-like domain composed of 20 residues with a length of ~35 Å (Fig. 2a). The overall structure of the cylindrical domain with a large central cavity is almost the same as that of hTIPE2^10^ (PDB ID: 3F4M) and hTIPE3^11^ (PDB ID: 4Q9V), indicating that the Tnfaip8 family shares a common structural motif (Fig. 3). The dimensions of the hydrophobic cavity are ~20 Å in depth and ~7 Å in diameter at the bottom up to ~10 Å at the entrance (Fig. 2b). The cavity is lined with highly conserved hydrophobic residues. Only a few prominently differing residues comprising the cavities are found among the three crystal structures. Leu109 of mTnfaip8 is substituted with Gly97 and Thr203 in hTIPE2 and hTIPE3, respectively. Leu51 of mTnfaip8 is replaced by Phe145 in hTIPE3 (Fig. S1). The volume of the cavity of mTnfaip8 is 837 Å^3^, and those of hTIPE2 and hTIPE3 are 822 Å^3^ and 805 Å^3^, respectively. Therefore, hydrophobic cofactors or substrates are expected to bind inside the cavity. Intriguingly, two long electron densities were found inside the cylindrical domain of mTnfaip8 (Fig. 2c). hTIPE2 and hTIPE3 also contain similar densities in the cavities of their cylindrical domains, as discussed below. In contrast, the electrostatic potentials of the outer surfaces of the Tnfaip8 family are degenerate (Fig. 4). Specially, a positively charged potential is prominently distributed from the surface of the grip-like domain straight down to the bottom of the cylindrical domain.

**Table 1:** Data collection statistics and refinement parameters for mTnfaip8.

	Tnfia8 (PDB code 5JXD)
**Data collection**	
Space group	I222
Cell dimensions	
*a*, *b*, *c* (Å)	66.28, 71.57, 90.60
*α*, *β*, *γ* (°)	90, 90, 90
Resolution (Å)	33.14–2.03 (2.07–2.03)*
*R*_sym_ or *R*_merge_	7.8 (86.9)
Volume fraction of solvent (%)	49.03
V_m_ (Å^3^/Da)	2.41
*I* / σ*I*	23.2 (1.6)
Completeness (%)	97.4 (74.8)
Redundancy	6.7 (4.8)
**Refinement**	
Resolution (Å)	33.14–2.03
No. reflections	13998
*R*_work_/*R*_free_	0.237/0.287
No. atoms	
Protein	1519
Ligand/ion	49
Water	21
*B*-factors	
Protein	52.8
Ligand/ion	66.1
Water	43.0
R.m.s. deviations	
Bond lengths (Å)	0.008
Bond angles (°)	1.54
**Ramanchandran plot**	
Residues in favored regions (%)	96.6
Residues in allowed regions (%)	3.4
Residues in disallowed (%)	0.0

*Values in parentheses are for highest-resolution shell.

### Identification of PE as a Tnfaip8-bound phospholipid

**Figure 5:**
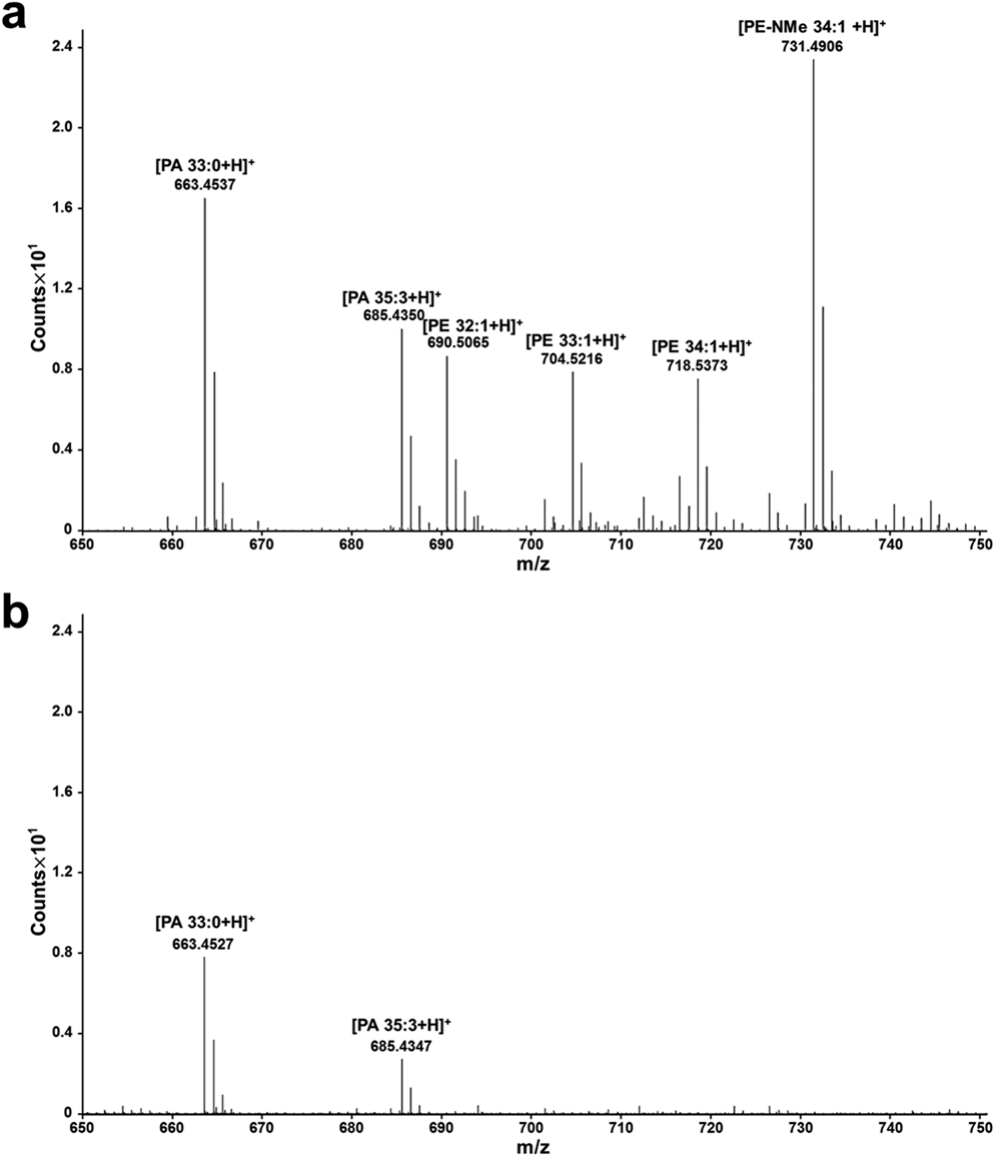
The results of LC-MS. LC-MS spectra of lipid extraction solutions from (**a**) mTnfaip8 and (**b**) the mTnfaip8 triple mutant.

LC-MS analyses were employed to identify candidate molecules fitting into the electron densities of the hydrophobic cavity of mTnfaip8. These analyses using a lipid extraction solution revealed a number of molecules, such as fatty acids (FAs) and phosphoglycerides. Because the mTnfaip8 protein used was overexpressed in *E*. *coli*, the electron density map inside the cavity may comprise average electron densities of a mixture of all bound FAs and phosphoglycerides. Among phosphoglycerides, PE with various FA chains were abundantly present in the solution. The major PE molecules detected can be grouped into saturated (30:0) and monounsaturated (32:1, 33:1, 34:1) (Fig. 5a). Considering the two long electron densities, the major components may be two FAs or one PE. To infer a preferred ligand and its molecular function, several candidate molecules were modeled into the electron density map and energy-minimized. Palmitic acid and oleic acid were suggested as the most plausible FAs. As the most promising PE candidate, PE 34:1 was selected and modeled into the cavity because the known major FA compositions of PE from *E*. *coli* are C_16:0_ (36%) and C_18:1_ (31%)^12^. In both cases, the FA portions were fit well in the electron density map. However, the PE-modeled structure provided more significant structural speculation. The PE-bound model explained a potent functional role of the three positively charged residues, H86, R87, K103, near the entrance of the cavity (Fig. 2a). These amino acids are next to the phosphate moiety of PE. However, the carboxylic groups of the modeled FAs are not located in the interaction distance of these residues. To confirm whether the three positively charged residues of mTnfaip8 actually influence PE binding, these residues were mutated to a negatively charged glutamate. LC-MS data with lipid extraction from the purified mutant clearly showed that much of the phosphoglyceride content was severely reduced (Fig. 5b). This result suggests that charge repulsion between the mutated glutamate residue and the phosphate moiety of PE eventually occurred and thus destabilized the binding of PE with mTnfaip8. However, FA molecules were less influenced by three residues and were still well detected.

### Critical role for the PE component in the anti-autophagic activity of mTnfaip8

**Figure 6:**
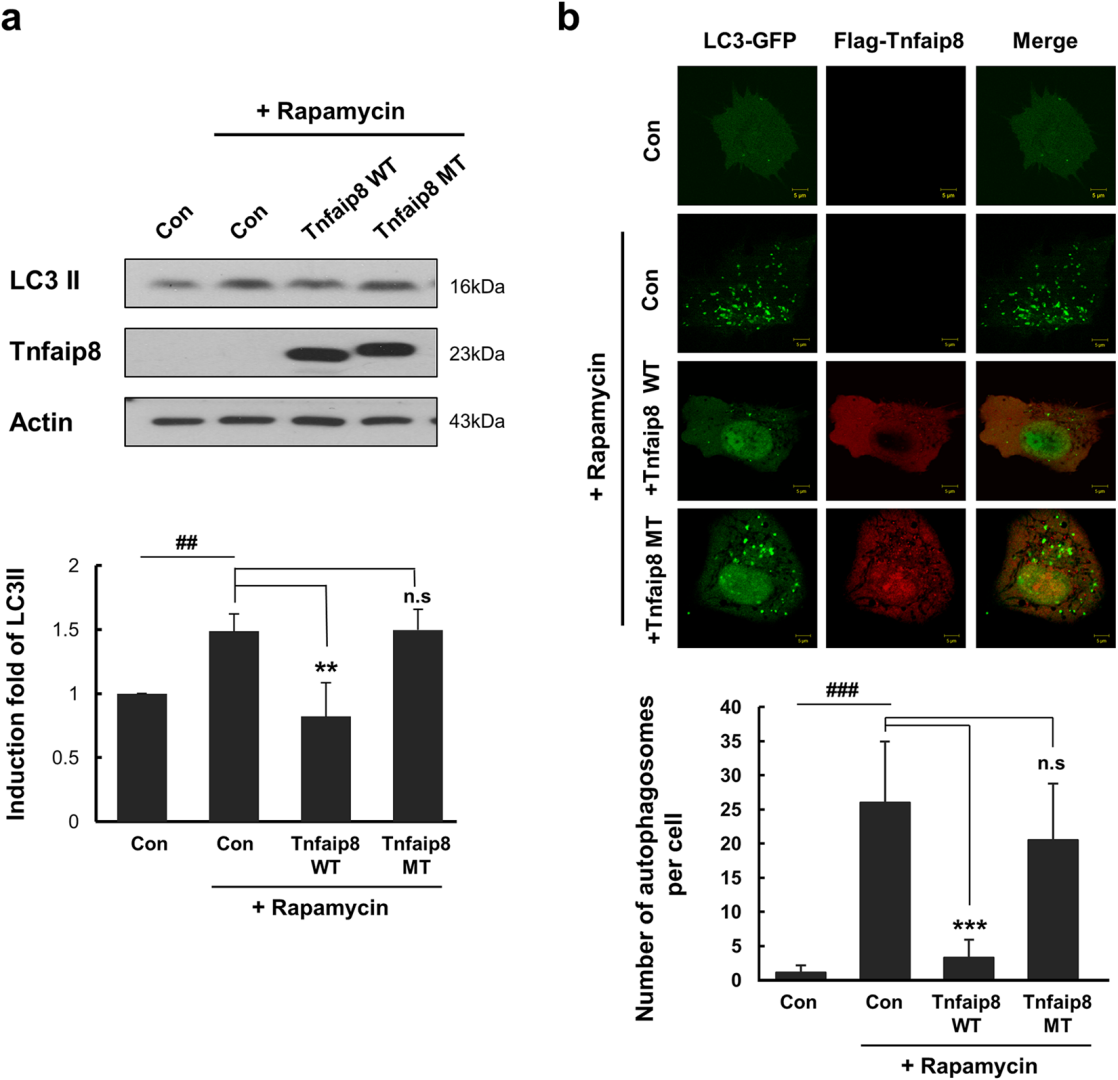
The anti-autophagic action of mTnfaip8. The anti-autophagic activities of mTnfaip8 (WT) and the mTnfaip8 triple mutant (MT) were assessed by analyzing the levels of autophagy marker LC3 II (**a**) and by quantitative confocal image analyses of GFP-LC3 puncta formation (**b**) in Hepa 1-6 cells after induction of autophagy by rapamycin (1 μM) for 3 h. The triple mutant (MT) dramatically lost inhibitory regulation of rapamycin-induced autophagy, whereas wild type (WT) demonstrated normal autophagic inhibition. Values are means (SD). ^##^p/**p < 0.01 and ^###^p/***p < 0.001. The full-length blots are presented in Supplementary Figure 3 (Fig. S3).

The crystal structure and LC-MS analyses revealed that mTnfaip8 is a novel PE-containing protein, in which PE is bound in the central cavity. Because PE is a crucial lipid component of the autophagosome and of phagophore expansion, which is provided by the PE-lipidated LC3 protein (i.e., LC3 II) during autophagy^13–15^, we examined whether the PE component of the mTnfaip8-PE complex exerts any physiological significance in anti-autophagy by employing a rapamycin-induced autophagy model^7^. We first generated a triple mutant of mTnfaip8 (H86E, R87E, K103E), in which the three positively charged residues near the entrance of the cavity were replaced by negatively charged glutamate residues and subsequently lost intrinsic PE-binding capacity (Fig. 5). As expected, the mTnfaip8 triple mutant dramatically lost inhibitory regulation of rapamycin-induced autophagy, whereas wild-type mTnfaip8 maintained the appropriate inhibitory regulation of autophagy, as demonstrated by immunoblotting (Fig. 6a) and confocal imaging (Fig. 6b). These results indicate an essential regulatory role for PE in the activity of mTnfaip8.

### PE deficiency in the hydrophobic cavity of the mTnfaip8 mutant resulting in defective coupling with Gαi3

**Figure 7:**
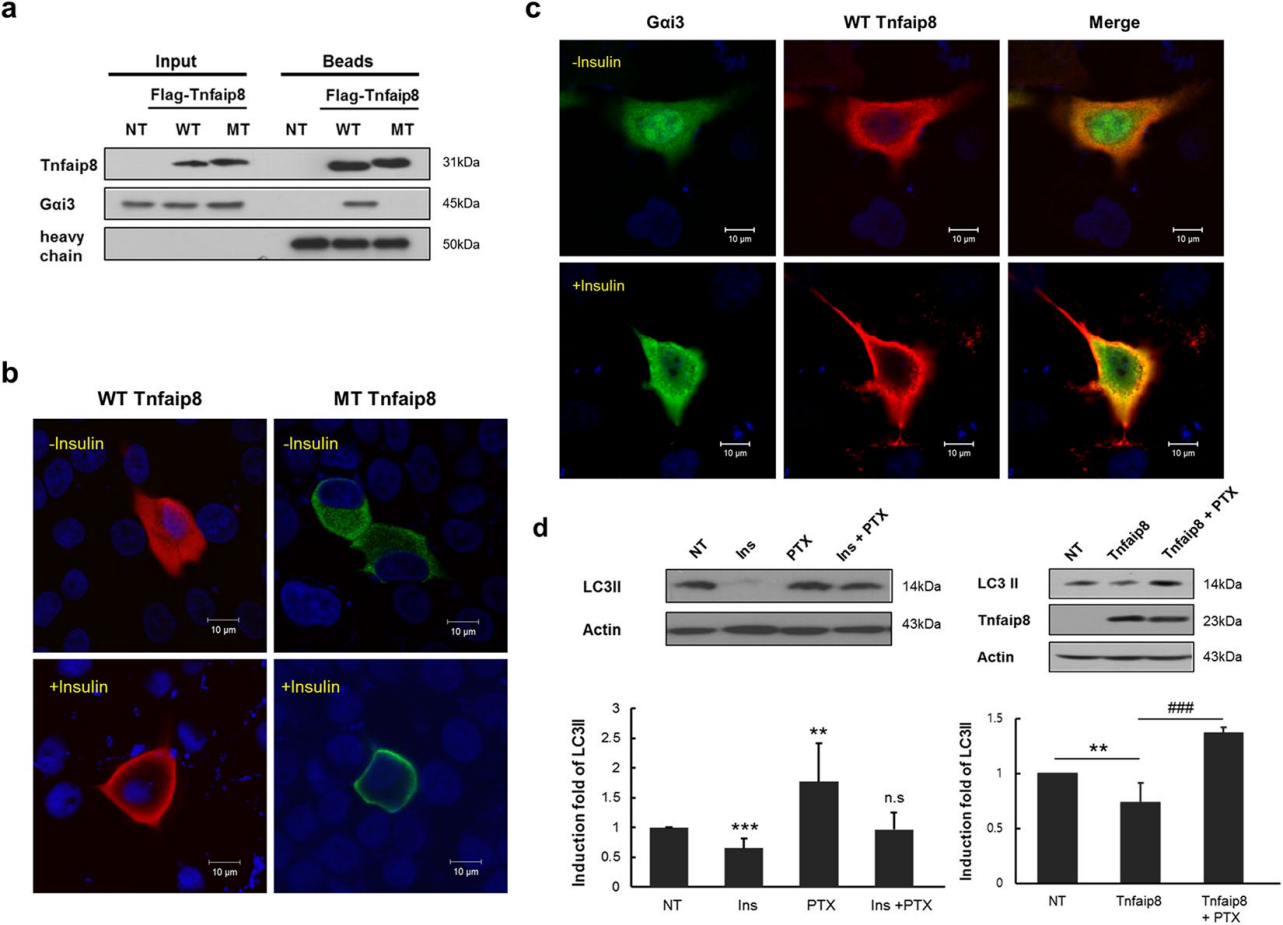
Interaction between wild-type mTnfaip8 and the triple mutant with Gαi3. (**a**) Co-immunoprecipitation assay using Flag-tagged wild-type mTnfaip8 (WT) and the Flag-tagged mTnfaip8 triple mutant (MT) was performed in Hepa 1-6 cells as described in Materials & Methods. As expected, the mTnfaip8 triple mutant with PE deficiency barely interacted with Gαi3, whereas wild-type mTnfaip8 maintained normal interaction with Gαi3. (**b**) HepG2 cells were transfected with Flag-mTnfaip8 (WT, red) and Flag-mTnfaip8 triple mutant (MT, green), followed by insulin treatment. Their subcellular relocalization was monitored by confocal microscopy. Representative images are shown here. Both cytoplasmic wild-type and mutant Tnfaip8 appeared to be relocated very significantly to plasma membrane by insulin treatment. (**c**) HepG2 cells were transfected with Flag-mTnfaip8 (WT, red) and myc-Gαi3 (green), followed by insulin treatment. The prominent relocation of both cytoplasmic mTnfaip8 and Gαi3 to plasma membrane after insulin treatment was observed. Representative images are shown here. (**d**) Treatment of Pertussis toxin (PTX) (100 ng/ml) was able to inhibit significantly insulin-induced anti-autophagy as demonstrated by the increased levels of LC3II (left panel). Moreover, anti-autophagic effect of Tnfaip8 overexpression was also inhibited by the PTX treatment (right panel). Values are means (SD). **p < 0.01 and ^###^p/***p < 0.001. The full-length blots are presented in Supplementary Figure 4 (Fig. S4).

Loss-of-function mutation of Gαi3 leads to defective anti-autophagic activity by insulin^3^. Furthermore, Gαi3 utilizes mTnfaip8 as an effector during cell death and oncogenic transformation *via* an unknown mechanism^16^. Therefore, the mechanistic significance of the mTnfaip8-PE complex was further investigated by addressing whether the Tnfaip8 mutant lacking PE retained its ability to couple with Gαi3 and then their subcellular distributions were examined during insulin-induced anti-autophagy. Indeed, the mTnfaip8 mutant with PE deficiency lost this Gαi3 coupling interaction, whereas the wild type maintained normal interaction with Gαi3 (Fig. 7a). However, both cytoplasmic WT and mutant Tnfaip8 appeared to be relocated very significantly to plasma membrane by insulin treatment (Fig. 7b), indicating Tnfaip8 mutant without PE still maintained the functional capability to relocate to plasma membrane by insulin treatment. We also observed the prominent relocation of both cytoplasmic Tnfaip8 and Gαi3 simultaneously to plasma membrane after insulin treatment (Fig. 7c). Finally, we further confirmed the interaction of mTnfaip8 with the active GTP form of Gαi3 by the abolishment of Tnfaip8-induced anti-autophagy via Pertussis toxin (PTX) treatment, which facilitated the ADP-ribosylation of Gαi3 (i.e. the inactive Gαi3) (Fig. 7d). These results further support the PE lipid component in the hydrophobic cavity of mTnfaip8 playing a critical functional role in its coupling with the active Gαi3 in plasma membrane by insulin treatment and that the mTnfaip8-PE complex acts as an essential upstream effector during insulin-induced anti-autophagy.

## Discussion

We have identified novel structural and functional mechanisms underlying upstream effector complex formation during the anti-autophagic action by insulin, of which dysregulation underlies insulin resistance. Our data demonstrate that insulin induces mTnfaip8, which is identified as a novel PE-containing protein, as shown in the PE-modeled crystal structure of mTnfaip8. Specifically, the overall structure of mTnfaip8 contains a cylindrical domain with a large central cavity, a common structural motif shared by the Tnfaip8 family members. Moreover, LC-MS analyses followed by molecular modeling of candidate phosphoglycerides revealed that PE is best accommodated in the electron densities of the mTnfaip8 hydrophobic cavity. Furthermore, we provided some evidences that the PE-binding ability of mTnfaip8 is essential for its anti-autophagic regulatory function *via* interaction most likely with Gαi3, which further implicates the mTnfaip8-PE-Gαi3 ternary complex as a potential novel therapeutic target to counteract the hepatic insulin resistance observed in obesity and diabetes.

In the X-ray crystal structure of mTnfaip8, the central hydrophobic cavity is connected to an N-terminal grip-like domain of which the function is unknown. The X-ray crystal structures of the N-terminal domains of mTnfaip8 and hTIPE3 have been determined, though that of hTIPE2 was not determined due to its extreme flexibility. The high average B-factors of their N-terminal domains indicate that they are intrinsically flexible due to the presence of a short hinge motif between α0 and α1 (Fig. 2a). Superimposition of the crystal structures of mTnfaip8 and hTIPE3 indicates that the N-terminal domain of mTnfaip8 is ~20° less deviated from the cylindrical domain than that of hTIPE3 (Fig. 2a). This deviation appears to largely originate from different crystallographic packing. Our structural analysis suggests that the flexibility of the N-terminal domain together with the described positively charged surface (Fig. 4) may be important for the biological functions of mTnfaip8 and hTIPE3 probably assisting in the uptake and release of phosphoglycerides.

Inside the cylindrical domain of mTnfaip8 (Fig. 2c), two long electron densities were found. Two unidentified, long electron densities were also reported in the hydrophobic cavities of hTIPE2 and hTIPE3, even though the densities were unmodeled in the refined crystal structures^10, 11^. LC-MS analyses of mTnfaip8 suggested that PE is the most populated substrate satisfying the electron densities inside the hydrophobic cavity as discussed in the Results section. Considering our PE-modeled crystal structure of mTnfaip8, the unmodeled electron densities of hTIPE2 and hTIPE3 are anticipated to be phosphoglycerides. A recent study showed that TIPE3 acts as the transfer protein for the lipid second messengers such as phosphatidylinositol 4,5-bisphosphate and phosphatidylinositol 3,4,5-trisphosphate, which promotes cancer^11^. The importance of this type of feature for phospholipid trafficking has been elucidated by multi-scale molecular dynamics simulations of an α-tocopherol transfer protein^17^. Therefore, the biological function of Tnfaip8 family members may also be connected to phospholipid trafficking. However, the presence of different amino acid sequences around the hydrophobic cavities, as discussed above, suggests that their preferred substrates may differ. For example, Leu51 of mTnfai8 inside the cavity is replaced by Phe145 in the case of hTIPE3, and H86 of mTnfaip8, which is located at the entrance of the cavity, is substituted by Tyr180 in hTIPE3 (Fig. 2a). Therefore, preferred types of head groups and lengths of FA moieties of phosphoglycerides may differ among Tnfaip8 family members and could render a differential biological function to each member of the Tnfaip8 family.

**Figure 8:**
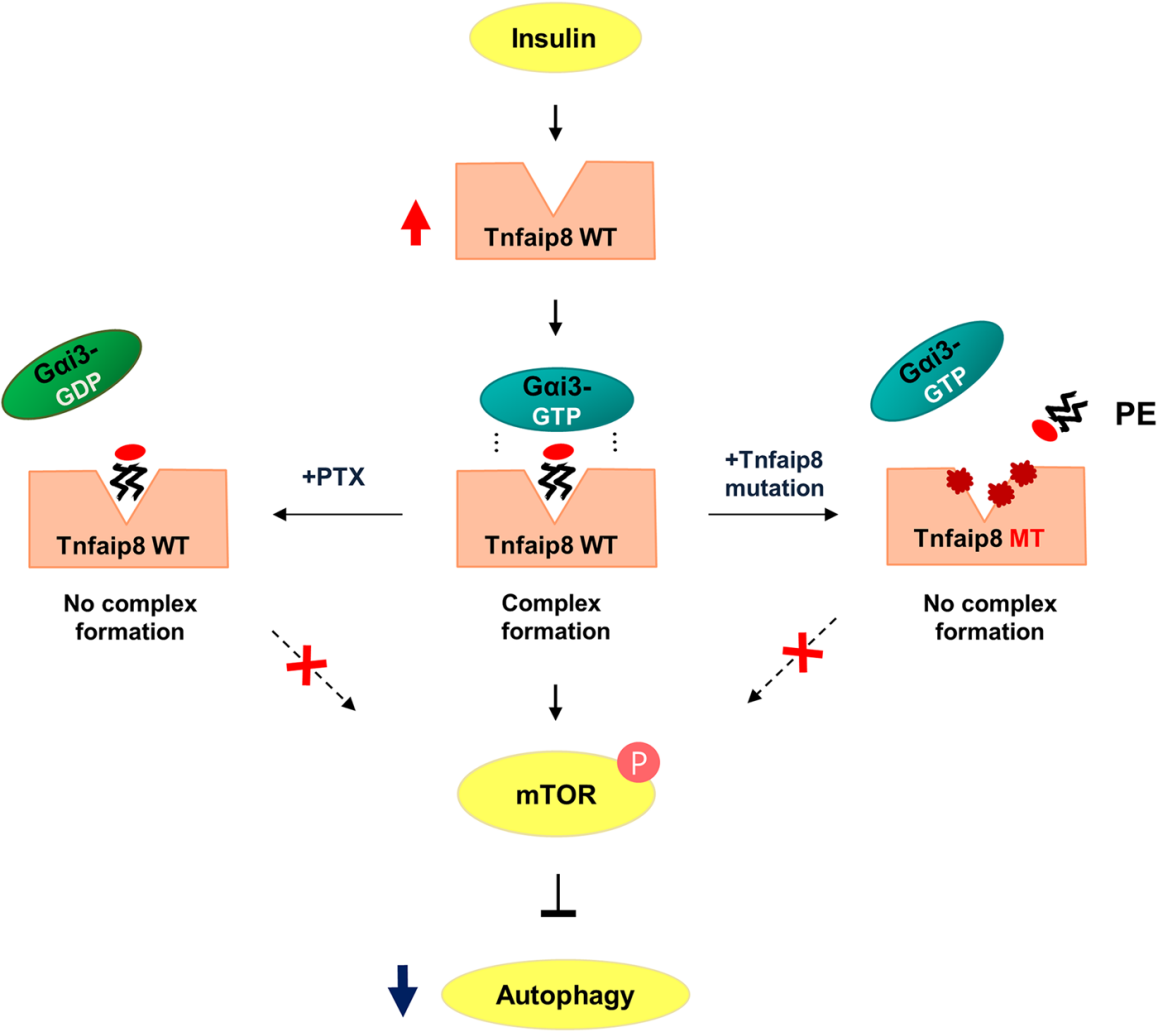
A schematic diagram for the anti-autophagic action of insulin. The anti-autophagic activity of insulin is mediated by upregulation of mTnfaip8, which is followed by the specific accommodation of PE in its hydrophobic cavity. The mTnfaip8 triple mutant defective for PE binding has lost intrinsic anti-autophagic capability, whereas the wild-type mTnfaip8 maintains anti-autophagic activity. PTX treatment also disrupted anti-autophagic activity of mTnfaip8 by accumulating the inactive GDP form of Gαi3, which has lost the coupling with mTnfaip8. The loss of anti-autophagic activity of the mTnfaip8 mutant is very likely due to impairment in coupling with the active GTP form of Gαi3.

Moreover, the PE molecule bound in the hydrophobic cavity appears to be essential for ternary complex formation with Gαi3, and the resulting ternary complex mTnfaip8-PE-Gαi3 mediates insulin-induced hepatic anti-autophagy, which further suggests a non-canonical role for Gαi3 in autophagy regulation under various physiological and pathological conditions, including insulin resistance (Fig. 8). In accordance with our findings, in previous studies, Gαi3 null mice exhibited defective insulin-induced anti-autophagy *via* an unknown mechanism and redistribution of Gαi3 into autophagosomes and the plasma membrane through autophagy induction^3, 4, 18^. Conventionally, heterotrimeric G proteins tightly associated with membranes transduce signals *via* G protein-coupled receptors (GPCRs)^19^. However, mounting evidence reveals non-canonical roles for G proteins, which regulate specific signaling pathways, such as integrinα_IIIB_β3 signaling, SRE activation and E-cadherin activity^20–22^ and function at subcellular organelles other than the plasma membrane, such as mitochondria, the Golgi and the nucleus^23–25^. Because the insulin receptor is not a GPCR, our data further suggest an important non-canonical signaling role for Gαi3 during insulin-induced anti-autophagy.

In conclusion, our data demonstrate that mTnfaip8 is a novel phosphoglyceride binding protein with PE as the most preferred substrate. PE binding in the hydrophobic cavity allows it to form a ternary complex with Gαi3, mTnfaip8-PE-Gαi3, and to mediate insulin-induced anti-autophagy. To date, no specific structural and functional features of the upstream effector complex, which mediates crosstalk between the insulin receptor and Gαi3-mTOR signaling during the hepatic anti-autophagic action of insulin, have been characterized. However, it may now be possible to improve hepatic insulin sensitivity through biochemical modulation of this novel ternary effector complex, which may help to circumvent the impaired hepatic autophagy occurring in insulin resistance. Moreover, our structural and functional characterization of the upstream effector complex may help in identifying a novel pharmacological target to counteract the hepatic insulin resistance observed in obesity and diabetes.

## Methods

### Structure determination of C165S mutant mTnfaip8

Details of the crystallization and preliminary X-ray study of C165S mutant mTnfaip8 have been previously published^26^. Since the wild type protein was easily precipitated during purification, C165S mutant was constructed to overcome the problem. Cys165 is located on the surface opposite of the ligand binding site and thus seems not to bother ligand binding. The brief summary of structure determination is as follows. The high-quality crystals of mTnfaip8 were obtained using 0.1 M Bis-Tris propane pH 7.7 and 1.2 M sodium citrate. The X-ray data were collected to 2.03 Å resolution. Based on the Matthews coefficient, the asymmetric unit could contain one protomer with a solvent content of 49.03% (V_M_ = 2.41 Å^3^ Da^−1^). Molecular replacement was performed using PHASER^27^ with the AutoMR wizard in PHENIX^28^. A relatively good electron density map was obtained and the initial model of mutant mTnfaip8 was well established with the PHENIX AutoBuild wizard^29^. The refinement of the initial model was performed using Coot^30^ and Phenix.refine^31^. The details of data collection and refinement statistics are shown in supplementary Table 1. The coordinates and structure factors have been deposited in the Protein Data Bank (PDB ID: 5JXD).

### Plasmids and Transient transfection

The expression plasmid pFLAG-CMV4-Tnfaip8 was constructed by the insertion of Tnfaip8 into a pFLAG-CMV plasmid with NotI and EcoRI digestion and used as a template for constructing a double mutant (H86E, R87E). The sequences of the mutagenic primers were 5′-GGTCATCAAGCTGGCCGTCCTCGAGGAGAACAATCAGTTCAATCAAGAC-3′ for the forward strand and 5′-GTCTTGATTGAACTGATTGTTCTCCTCGAGGACGGCCAGCTTGATGACC-3′ for the reverse strand. The plasmid DNA of the double mutant was used as a template for constructing a triple mutant (H86E, R87E, K103E). The sequences of the mutagenic primers for the triple mutant were 5′-CGCTCATGGAGAAGTTCGAGAAGAAGGTGCACCAG-3′ for the forward strand and 5′-CTGGTGCACCTTCTTCTCGAACTTCTCCATGAGCG-3′ for the reverse strand. The polymerase chain reaction (PCR) and transformation was carried out with a QuikChange Lightning Site-Directed Mutagenesis Kit (Agilent Technologies, La Jolla, California, USA) according to the manufacturer’s protocol. Transformed double and triple mutant plasmids were purified using DNA-spin plasmid DNA purification kit (iNtRON Biotechnology, Seoul, Korea). The vector expressing LC3-GFP was a kind gift from Dr. Katsuyoshi Mihara. For transient expression of plasmids, cells were cultured 24–36 h before transfection with Fugene 6 (Roche, Indianapolis, IN, USA) or Fugene HD (Promega, Madison, WI, USA).

### Gene cloning, protein expression and purification for the triple mutant

Cloning, expression and purification of the protein was carried out using previously reported method^26^. In brief, the amplified triple mutant was annealed into the amplified LIC expression vector pB2 and transformed into DH5α competent cells. Clones were screened by plasmid DNA analysis and transformed into *E*. *coli* BL21 (DE3) for protein expression. The transformed cells were grown at 310 K with shaking until the OD 600 nm reached at 0.6–0.8, and then incubated at 298 K for 6 h in a shaking with 1 mM isopropyl *β*-D-1-thiogalactopyranoside for expression. Cells were collected by centrifugation at 6,500 rpm for 10 min at 4 °C. The pellet was suspended and disrupted using a Digital Sonifier 450 (Branson Ultrasonics Co., Danbury, Connecticut, USA). Cell debris was centrifuged at 15,000 rpm for 30 min at 4 °C and the supernatant was filtered using a Millex-LCR syringe-driven filter (Millipore, MA, USA) with a pore size of 0.45 *μ*m. The triple mutant protein was purified on an ÄKTA Explorer system (GE Healthcare, Piscataway, New Jersey, USA). The filtered supernatant was affinity-purified using a HisTrap column (GE Healthcare) equilibrated with a buffer consisting of 50 mM Tris–HCl pH 8.0, 300 mM NaCl and 10 mM imidazole. The target protein was eluted with a buffer consisting of 50 mM Tris–HCl pH 8.0 and 100 mM NaCl with a gradient from 10 to 500 mM imidazole. The pooled fractions were loaded onto a 5 mL Hi-Trap heparin HP column (GE Healthcare) equilibrated in 20 mM Tris pH 8.0 and eluted with a gradient from 0 to 2 M NaCl. Further purification by gel filtration chromatograph was performed using a Superdex 200 10/300 GL gel filtration column (10 × 300 mm) with 20 mM Tris pH 8.0.

### Phospholipid extraction from protein

The extraction of phospholipids was performed using a single-phase extraction method^32^. In order to extract phospholipids from protein, 50 *μ*L of protein solution was mixed with 1 mL of ice-chilled solution of chloroform-methanol (1:1, *v*/*v*), followed by incubating at 4 °C for 30 min. The mixture was centrifuged at 13,000 rpm for 15 min at 4 °C. After collecting the supernatant, the pellets were resuspended in 1.5 ml of chloroform-methanol (2:1, *v*/*v*). The resuspended solution was incubated at 4 °C for 30 min and centrifuged at 13000 rpm for 15 min at 4 °C. The pooled centrifuged supernatants were evaporated under nitrogen and re-dissolved in 1 mL of chloroform-methanol-water (50:45:5, *v*/*v*).

### Liquid chromatography-mass spectrometry (LC-MS)

10 *μ*L of the prepared sample containing phospholipid was injected into an Agilent 1200 HPLC system (Agilent). 50% (*v*/*v*) of water with 0.1% (*v*/*v*) formic acid and 50% (*v*/*v*) acetonitrile with 0.1% (*v*/*v*) formic acid was used as an isocratic mobile phase at a flow rate of 0.4 mL/min. The HPLC system was connected to an Agilent 6220 Accurate-Mass TOF (Agilent) equipped with an electrospray interface operating in positive ion mode. The optimized operation parameters were as follows: capillary voltage, 4000 V; nebulizer pressure, 30 psi; drying gas flow rate, 11 L/min; gas temperature, 673 K; skimmer voltage, 60 V; octapole rf, 250 V; fragment voltage (in-source CID fragmentation), 200 V. LC-MS accurate mass spectra were recorded across the range m/z 50–1000. The accurate-mass calibration was performed over a mass range of m/z 118.0863–2721.8950 using a calibration solution provided by the manufacturer (G1969-85000, Agilent, Santa Clara). The full-scan data were recorded with the Agilent Mass Hunter Data Acquisition software (version B.03.01) and processed with the Agilent Mass Hunter Qualitative Analysis software (version B.03.01).

### Cell cultures, Chemicals and Antibodies

HEPA 1–6 cell line and HepG2 cells were cultured as recommended by the American Type Culture Collection (Manassas, MA, USA). Rapamycin and Insulin were purchased from Sigma-Aldrich (St.Louis, MO, USA). Pertussis toxin was purchased from Calbiochem (San Diego, CA, USA). Antibodies for LC3, p-PI3K p85 (Tyr458)/PI3K, p-Akt/Akt, and p-mTOR/mTOR were from Cell Signaling (Danvers, MA, USA). Antibodies for Gαi3 and β-actin were from Santa Cruz Biotechnology (Dallas, TX, USA). Antibodies for Tnfaip8 were both produced using protein-specific synthetic peptides from AbFrontier (Seoul, South Korea) as described before^7^ and purchased from Proteintech (Rosemont, IL, USA).

### Western blot and Immunoprecipitation analysis

As previously described^7^, cells grown under experimental conditions were washed with ice-cold phosphate-buffered saline (PBS) and lysed for 30 min on ice in radioimmunoprecipitation assay (RIPA) buffer containing protease inhibitor cocktails and phosphatase inhibitors (Roche, Indianapolis, IN, USA). Cells were scraped on ice, then, centrifuged at 14,000 rpm for 20 min at 4 °C. Protein concentrations were determined by the Bradford method using bovine serum albumin (BSA) as the standard. After denaturation with 5× sample buffer and boiling at 90 °C for 5 min, protein samples were separated by sodium dodecyl sulfate-polyacrylamide gel electrophoresis (SDS-PAGE) and transferred onto pre-wetted polyvinylidene fluoride (PVDF) membrane (Millipore, Billerica, MA, USA). The membranes were incubated with 5% non-fat dry milk or 5% BSA in Tris-buffered saline (TBS) with 0.1% Tween 20 to block non-specific binding. Primary antibodies were diluted in 5% non-fat dry milk or 1% BSA in TBS with 0.1% Tween 20 and incubated overnight at 4 °C with gentle shaking. Blots were probed with proper secondary antibodies and visualized by enhanced chemiluminescence (ECL) (Amersham Biosciences, GE Healthcare, Buckinghamshire, UK). Membranes were exposed to X-ray films (AGFA, Greenville, SC, USA), and densitometric quantification of the immunoblotted membranes was performed with an Image Analyzer system using Multi Gauge version 2.3 software (Fujifilm, Tokyo, Japan). For immunoprecipitation analysis, cells were transfected and lysed in the M-PER mammalian extraction buffer (Pierce, Rockford, IL, USA) containing protease inhibitor cocktail (Roche, Indianapolis, IN, USA) for 30 min on ice. Cells were scraped on ice, centrifuged at 14,000 rpm for 20 min at 4 °C. Lysates were incubated with Flag-specific mAb M2 coupled agarose beads (Sigma-Aldrich) overnight at 4 °C in Phosphate-buffered saline (PBS), pH 7.4. After incubation, the beads were washed with PBS and boiled in 5× sample buffer for 4 min, and analyzed by Western blot.

### Confocal microscopy

Cells were cultured on poly-l-lysine coated two-well slides or cover slides and transfected with plasmids. After incubation and chemical treatment, the cells were fixed with 4% paraformaldehyde for 20 min and washed with PBS. Immunolabeling followed blocking with 1% BSA in PBS with 0.1% Triton-X 100. After primary antibody incubation, the cells were labeled with appropriate Alexa Fluor secondary antibodies. The culture slides were mounted with Vectashield aqueous mounting medium (Vector Laboratories, Burlingame, CA, USA) and analyzed on a Zeiss LSM510 META laser scanning confocal microscope (Carl Zeiss, Germany). Image processing and analysis were performed using Zeiss LSM510 software version 2.36.

### siRNA and gene knockdown

The Tnfaip8 gene-specific siRNA and negative control siRNA duplexes were purchased from Bioneer (Seoul, South Korea). The sequence of sense siRNA oligonucleotide is 5′-CUCAGAGCUAGUGGCA UGA-3′. For gene knock down, the siRNA duplexes (100 pmoles/well for 6 well plate) were delivered using the Lipofectamine RNAiMAX transfection reagent (Thermo Fisher, Waltham, MA, USA) to Hepa1-6 cells 48 h before cells were harvested.

### Statistical analysis

For statistical analyses, two-sample comparisons were performed with Student’s t-tests, and multiple comparisons were performed using one-way analysis of variance (ANOVA) followed by appropriate post hoc comparisons.

## Electronic supplementary material

Supplementary figures
