# Supplementary material for: The Tnfaip8-PE complex is a novel upstream effector in the anti-autophagic action of insulin
Source: Sci Rep. 2017 Jul 24;7:6248. doi: 10.1038/s41598-017-06576-3 (PMC5524748; doi:10.1038/s41598-017-06576-3)

The Tnfaip8-PE complex is a novel upstream effector in the anti-autophagic action of insulin

Ji-Soo Kim¶, Jimin Park¶, Mi-Sun Kim¶, Ji-Young Ha, Ye-Won Jang, Dong Hae Shin* and Jin H. Son*

College of Pharmacy and Graduate School of Pharmaceutical Sciences, Global Top5 Research Program, Ewha W. University, Seoul 120-750, Republic of Korea

Supplementary Fig. 1

The superimposed cavity structure of mTnfaip8 (green) with that of hTIPE2 (gold; PDB ID: 3F4M) and hTIPE3 (sky blue; 4Q9V)

Different residues comprising the cavities are drawn with a stick model and labeled according to their ribbon colors.

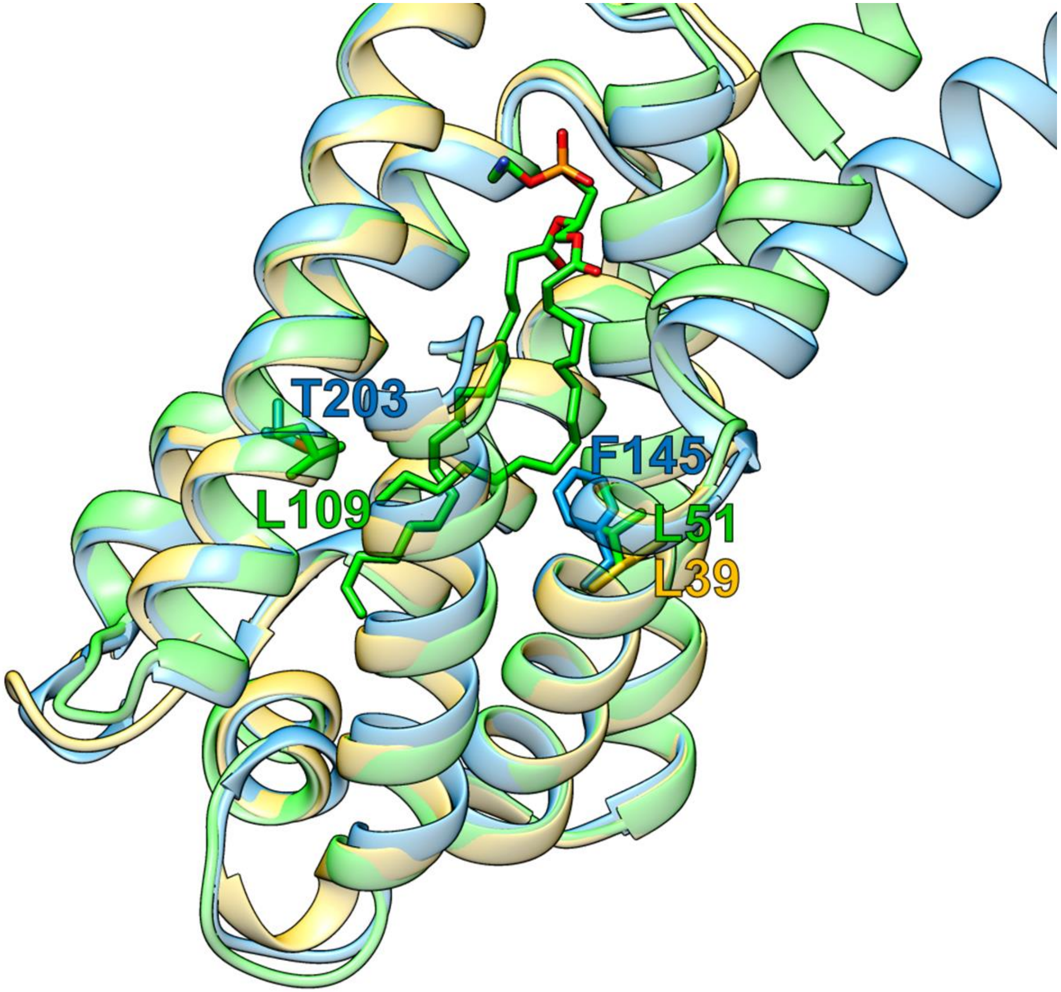

Supplementary Fig. 2 (For Fig. 1)

Temporal induction of mTnfaip8 by insulin

(**Fig 1-a**) The anti-autophagic activity of mTnfaip8 was confirmed in a hepatic cell line.
mTnfaip8 was induced by insulin, followed by phospho-mTOR upregulation and LC3-II downregulation.

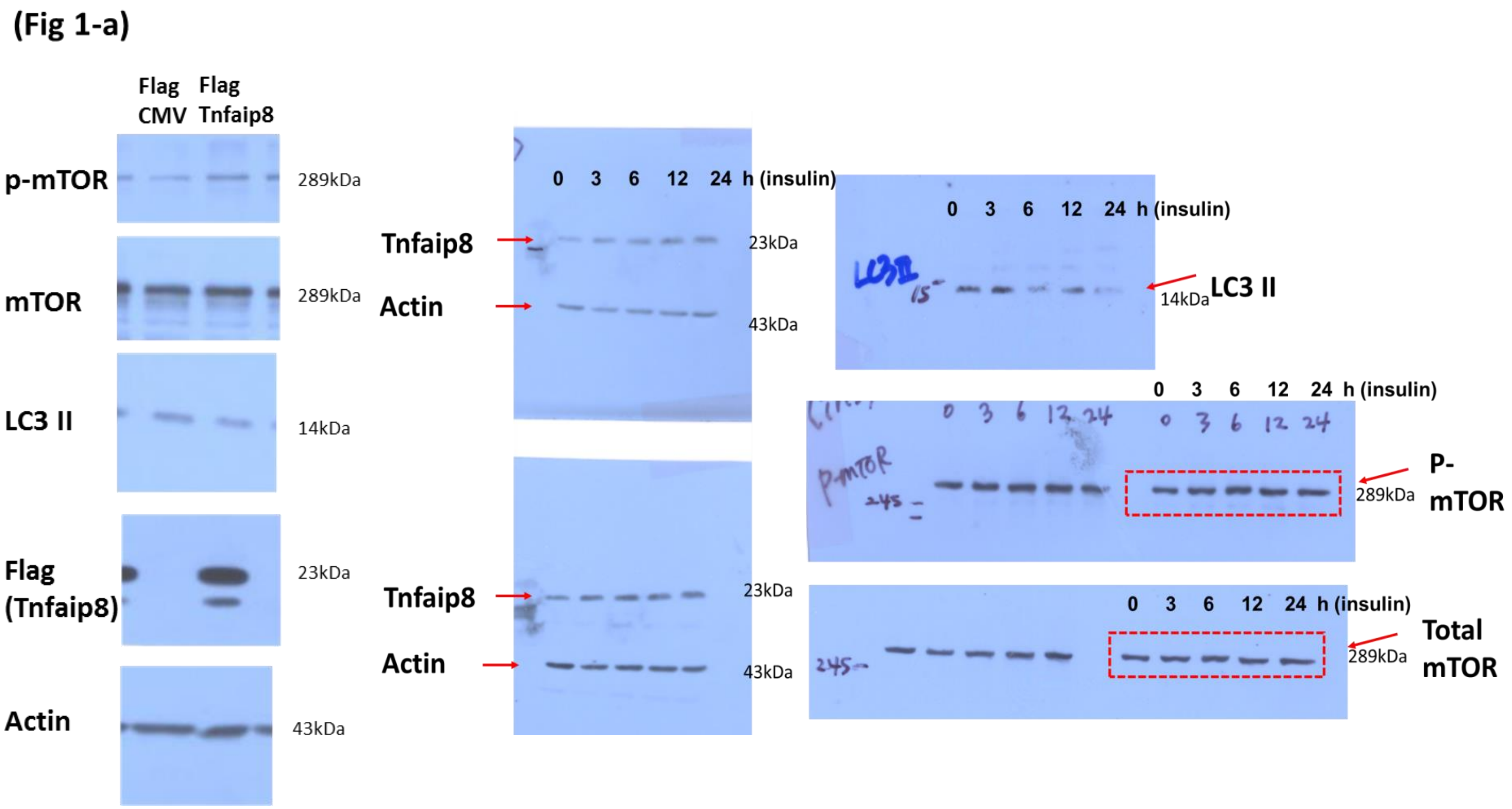

(**Fig 1-b/c**) The levels of phospho-PI3K, phospho-Akt and phospho-mTOR were decreased and the induction of autophagy was observed in both the absence and presence of insulin

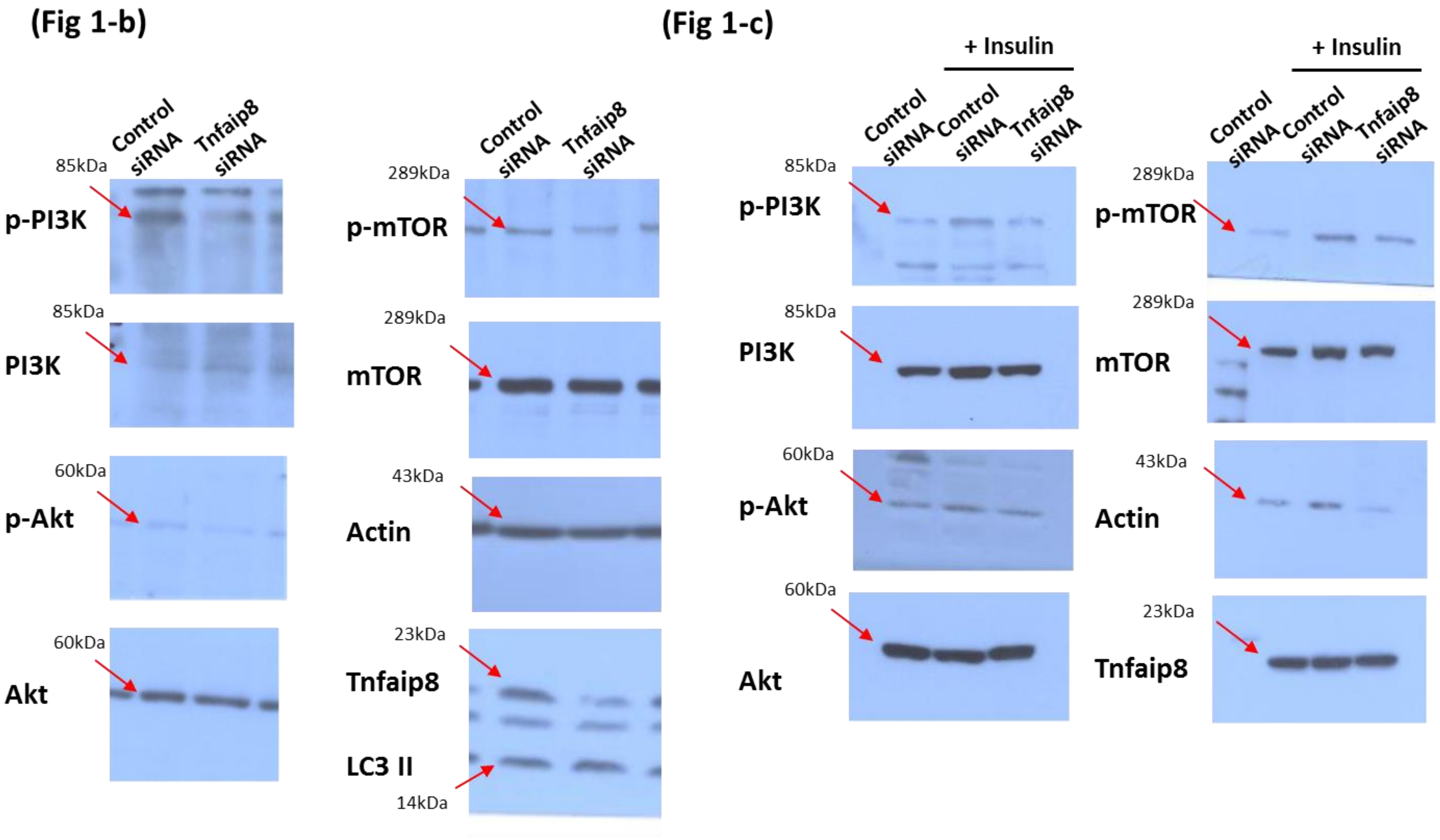

Supplementary Fig. 3 (For Fig. 6)
The anti-autophagic action of mTnfaip8

The triple mutant (MT) lost inhibitory regulation of rapamycin-induced autophagy, whereas wild type (WT) demonstrated normal autophagic inhibition

(Fig 6-a)

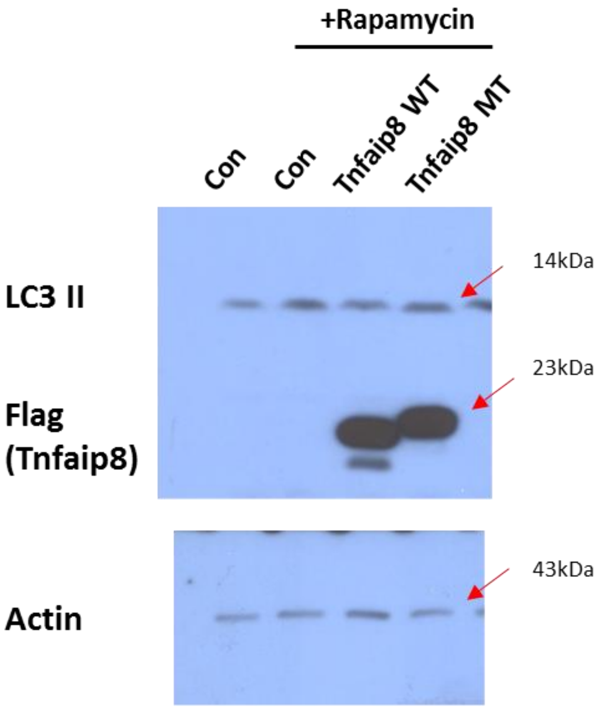

Supplementary Fig. 4 (For Fig. 7)

Interaction between wild type mTnfaip8 and the triple mutant with Gαi3

(**Fig 7-a**) The mTnfaip8 triple mutant with PE deficiency barely interacted with Gαi3, whereas wild type mTnfaip8 maintained normal interaction with Gαi3.
(**Fig 7-d**) Pertussis toxin (PTX) (100 ng/ml) inhibited insulin-induced anti-autophagy. Anti-autophagic effect of Tnfaip8 was also inhibited by the PTX treatment.

(Fig 7-a)

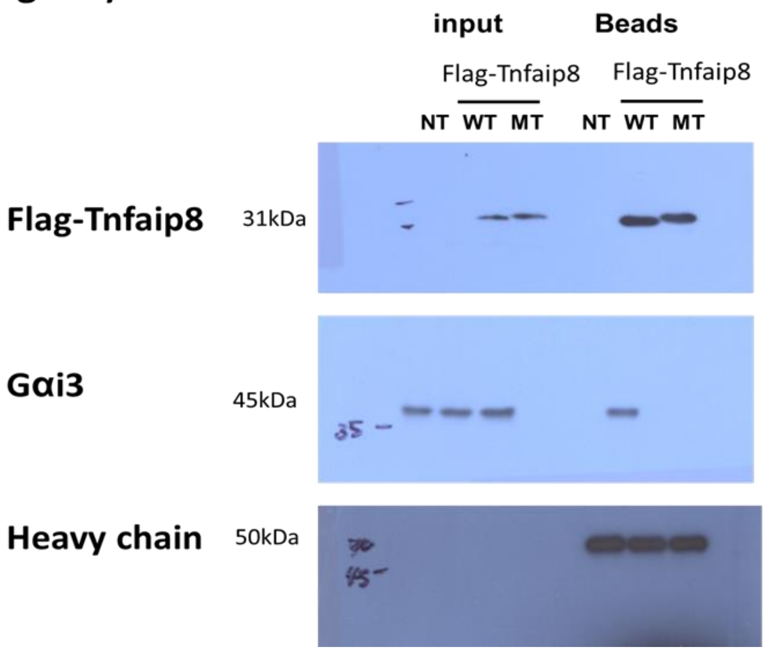

(Fig 7-d)

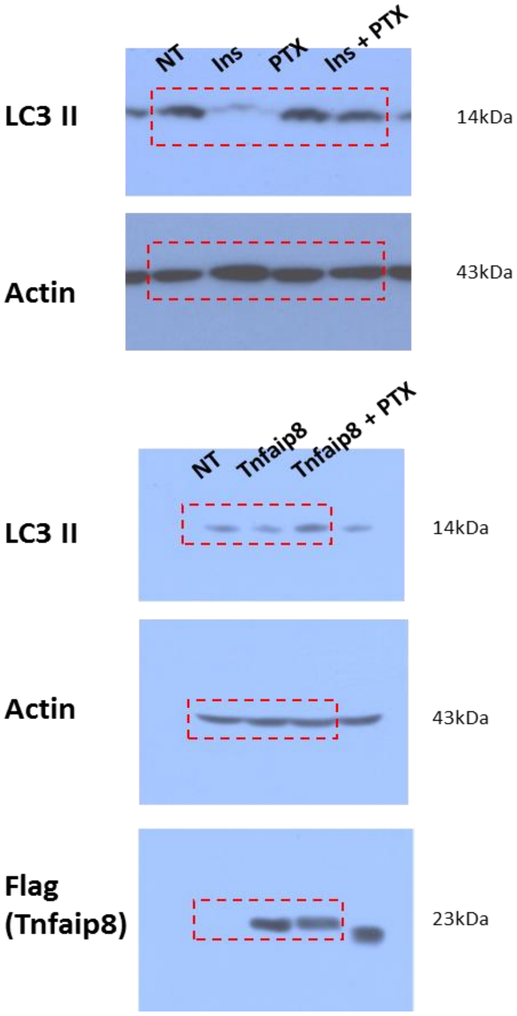

Supplement: Supplementary file 1 — Supplementary figures [file 41598_2017_6576_MOESM1_ESM.pdf]
